# Cerebral infarctions following an increase in corticosteroids: an atypical case of reversible cerebral vasoconstriction syndrome

**DOI:** 10.1007/s00415-022-11170-3

**Published:** 2022-05-16

**Authors:** Deborah Katharina Erhart, Albert Christian Ludolph, Katharina Althaus

**Affiliations:** Department of Neurology, University of Ulm, Oberer Eselsberg 45, 89081 Ulm, Germany

Dear Editors,

The causes of reversible cerebral vasoconstriction syndrome (RCVS) are manifold. Among the most common are the use of certain vasoactive drugs such as antidepressants, nasal decongestants, triptans and immunosuppressants (tacrolimus, cyclophosphamide) as well as illicit drugs (cannabis, amphetamines, ecstasy and cocaine), postpartum period, traumatic brain injury, catecholamine-producing tumors and vascular association (cervical artery dissection and carotid endarterectomy) [1]. Middle-aged women are most often affected. In 82–100% of cases, the first symptom is thunderclap headache (TCH) [2]. Common complications are ischemic stroke (39%), cortical subarachnoid hemorrhage (cSAH) (34%), intracerebral hemorrhage (ICH) (20%) and brain vasogenic edema consistent with posterior reversible encephalopathy syndrome (PRES) (38%) [3]. Often, ischemic strokes due to RCVS are located in border zones and bilaterally [4]. These consequences usually occur about 2 weeks after the TCH-episode [5]. The pathophysiology behind RCVS is poorly understood and multifactorial. It is thought to be due to an aberrant sympathetic response of vascular tone regulation starting distally and moving proximally [5]. One possible reason for the time lag between hyper-acute, worst-ever headache and stroke is the direction of vasoconstriction from small to large vessels [6].

In the following, we will present a case of a 67-year-old female patient who presented to our neurological hospital with cerebral ischemia each time following dose increase of oral prednisolone. Afterwards, we will discuss the specifics of the case.

We report on a female patient who has been intermittently admitted to our neurological hospital since October 2020 due to recurrent cerebral infarctions in the territory of the right anterior cerebral artery, each time after increasing oral low-dose prednisolone due to rheumatoid arthritis. Other medications included pantoprazole, tilidine/naloxone, a statin, non-steroidal anti-inflammatory drugs (NSAID) and adalimumab, a tumor-necrosis factor α inhibitor (TNF-α). Due to a heterozygous factor II mutation and a right–left shunt, the patient was already orally anticoagulated with a vitamin K antagonist as initially paradoxical embolisms were suspected. Even under this medication, the patient developed further cerebrovascular events. Cerebrospinal fluid (CSF) findings showed no evidence of an inflammatory genesis of the ischemic stroke (white-blood-cell count 2/µl, no elevated total protein levels, no isolated intrathecal IgG-synthesis). During the last inpatient stay, the patient developed twice sudden onset of left leg-stressed hemiparesis and aphasia in direct temporal relation to increase of prednisolone dosage due to an exacerbation of arthralgias. Immediate diagnostic investigation with MRI and time-of flight (TOF) angiography as well as computed tomography (CT) of the head revealed a marked perfusion delay in the territory of the right anterior cerebral artery (ACA) with partial vessel breaks or stenosis of the right ACA (Figs. 1, 2a–c). Vessel narrowing of the right vertebral artery was also detected, whereas the left vertebral artery was more intense (Fig. 2c). There was no evidence of perfusion disturbance in the posterior circulation. A color-coded duplex sonography of the right temporal artery showed no dark concentric vessel wall thickening. In a digital subtraction angiography (DSA) during the course, even previously detected vasoconstrictions could no longer be traced. Suspecting an RCVS, although recurrent severe headache attacks and TCH were denied, we started a therapy with the calcium antagonist nimodipine. Imaging control at later timepoints showed only relatively small or no diffusion disturbances and the vessel breaks and stenoses were regressed (Figs. 2d, 3). Regular transcranial Doppler (TCD) follow-up showed normal maximal mean flow velocities in the cerebral arteries and neither focal neurological deficits nor new ischemic lesions have occurred so far. A possible explanation for the additional intermittent aphasia could be a transcortical motor aphasia due to pronounced perfusion delay in the territory of the right ACA together with older infarcts in the territory of right middle cerebral artery in left-handed patient. Otherwise, further vasospasm in the Broca’s area cannot be ruled out. However, we do not have any evidence in imaging for this.

**Fig. 1:**
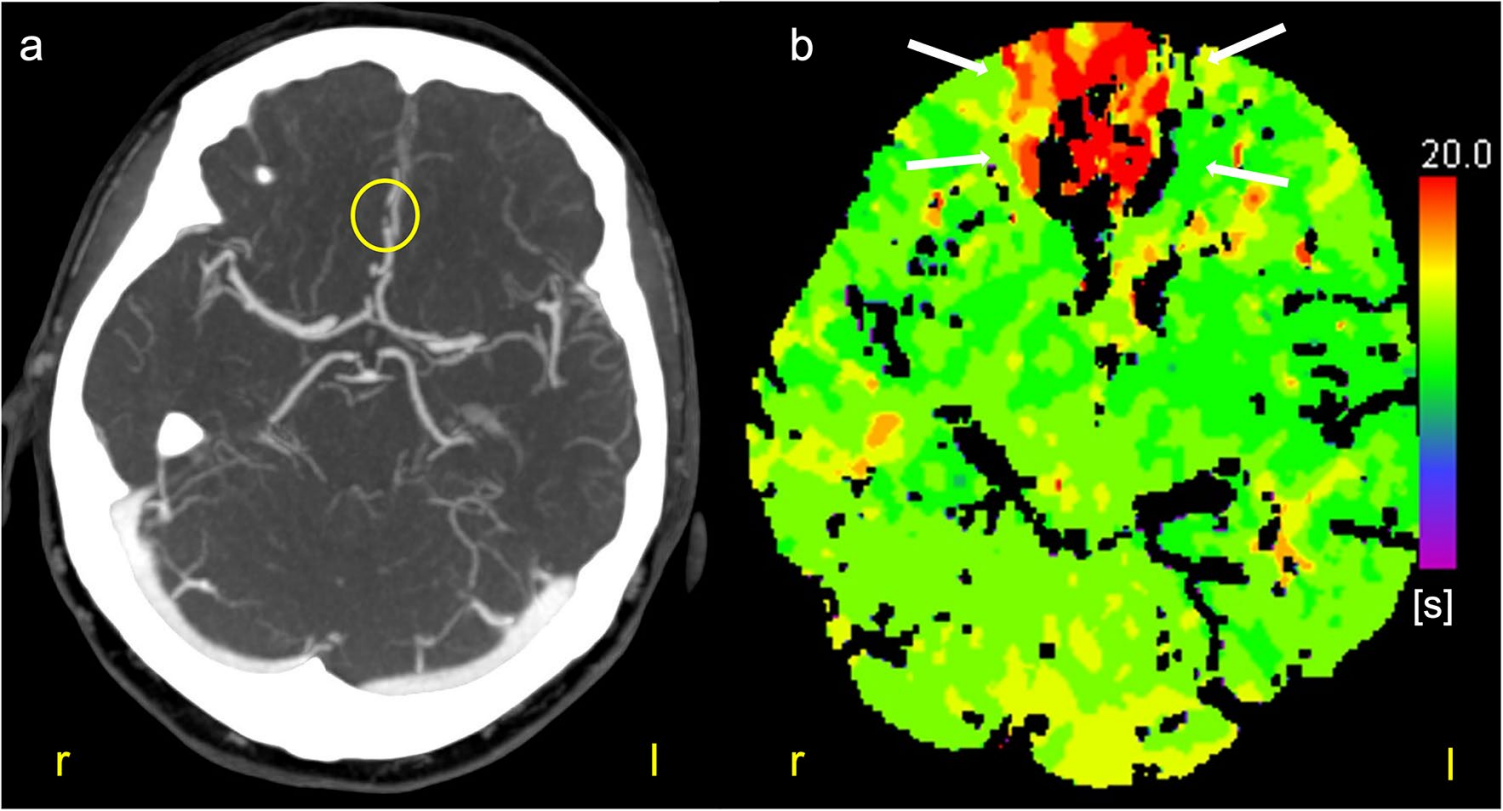
Various computed tomographic (CT) images of a 67-year-old female patient with reversible cerebral vasoconstriction syndrome (RCVS). CT-angiography after acute left leg-stressed hemiparesis (**a**). At the transition of A2-to-A3-segment of the right anterior cerebral artery (ACA), a short-stretch vessel stenosis is found, compatible with a vasospasm (yellow circle). In the anterior circulation (right ACA territory) (**b**), there is a frontal time-to-peak-delay (TTP) in the perfusion-CT (white arrows, s: second)

**Fig. 2:**
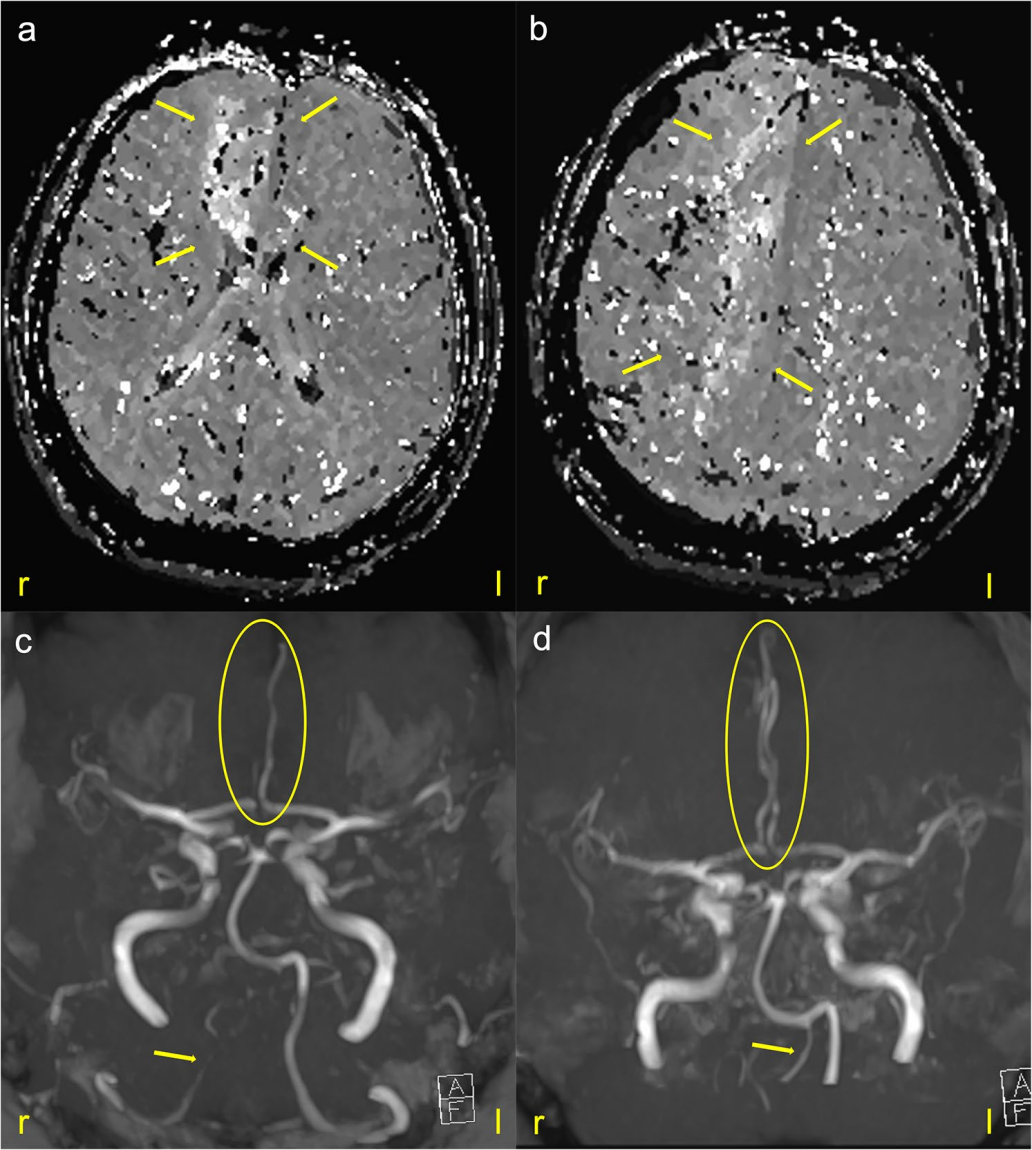
Magnetic resonance imaging (MRI) at a further timepoint. An acute leg-stressed left hemiparesis with aphasia appeared again. Perfusion-weighted imaging (PWI) showed extensive perfusion delay in the right anterior cerebral artery, marked with yellow arrows (**a**, **b**). In the MR-time of flight (TOF) angiography, the right anterior cerebral artery can no longer be visualized from the A2-section (**c**) (yellow ellipse). The right vertebral artery also shows only a weak signal (yellow arrow) resembling a vessel narrowing with an intense left vertebral artery. During intravenous administration of nimodipine, both vessels could be visualized again (**d**) with a regular flow profile (yellow ellipse and arrow)

**Fig. 3:**
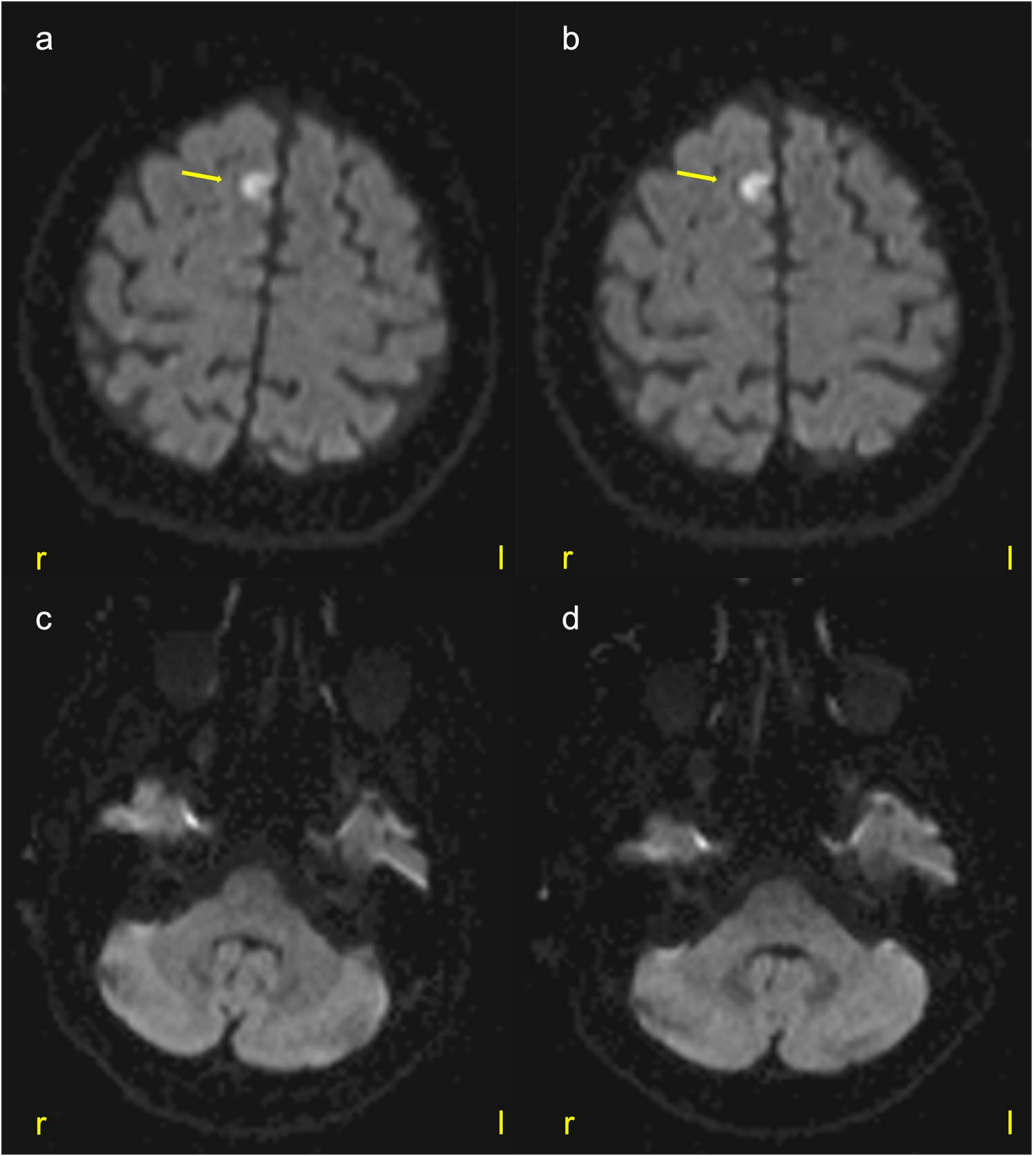
MRI findings of the brain parenchyma. Despite the extended perfusion delay in PWI (see Fig. 2a, b), only a small ischemic lesion can be detected in the right anterior territory (yellow arrow) in diffusion-weighted imaging (DWI). In the right posterior circulation (vessel narrowing of the right vertebral artery, see Fig. 2c), there was neither evidence of perfusion delay (not shown) nor any diffusion disturbance. (**a**, **c**) DWI at the time of PWI, (**b**, **d**) DWI 4 h later

Furthermore, the patient did not suffer any permanent neurological deficits. Prednisolone was tried to be phased out and recurrent arthralgias were transitionally treated with NSAID.

To the best of our knowledge, this is the first case report of the development of RCVS as a result of low-dose corticosteroid medication or dose increase. In total, the patient presented four times with cerebral ischemia in the anterior circulation due to an increase in oral low-dose prednisolone.

An important feature in our case is the absence of thunderclap headache (TCH). Hyperactive severe headache is a cardinal symptom of RCVS. Only in 0–15% of all cases is a course without TCH described [2]. Recurrent TCH is not only pathognomonic for RCVS. This leads to the most important differential diagnoses of RCVS: subarachnoid hemorrhage, cerebral venous sinus thrombosis, arterial dissection and pituitary apoplexy. Immediate cerebral imaging by CT or MRI is indicated to rule out these serious conditions [7]. Studies have shown that 55% of RCVS patients had no cerebral lesions on initial imaging [3].

Another important differential diagnosis in our case is primary angiitis of the central nervous system (PACNS). Characteristically, it is a dull, persistent headache instead of TCH, but neurovascular imaging with vessel breaks and various irregularities in different vessels may resemble RCVS [7]. An important distinguishing factor is the irreversibility of the vascular changes in PACNS, which is why intra-arterial calcium channel blockers are often tried diagnostically [7]. In our case, DSA in the further course showed no signs of vasospasm anymore.

Empirical immunosuppressive therapy with glucocorticoids is often administered to RCVS patients out of fear of overlooking PACNS or due to the misdiagnosis of PACNS [8]. To the date, there are only a few studies that have shown that glucocorticoid administration, regardless of the application form, is associated with a worse outcome, according to modified Rankin scale 4–6 (mRS) [3, 8, 9]. In subgroup analyses, further disease progression with clinical, radiological and angiographic deterioration could be detected in up to 48% [3]. Different from our case, approximately 60% of patients with glucocorticoid-induced worsening of RCVS had use of serotonergic medications. All of them were women [8]. Most RCVS patients (treated with calcium channel antagonists or without therapy) have a self-limiting course with good outcome [3]. Persistent neurological deficits occur in only 10% [4, 8]. Glucocorticoids do not prevent deterioration—on the contrary—they even lead to worsening by potentiating the vasoconstrictor effects of angiotensin II (ATII), norepinephrine and endothelin through upregulation of alpha1- and ATII-receptors. Thus, they lead to an increase in sympathetic activity [8, 10]. Likewise, glucocorticoids have direct effects on vascular smooth muscle cells by leading to their hyperplasia and hypertrophy [10].

Due to the reversibility of vasoconstrictions in the anterior and posterior circulation, the relatively small established ischemia after marked frontal perfusion delay, no evidence of vasospasm in conventional angiography during the course and the lack of inflammatory CSF findings, which occur in up to 90% of PACNS-patients, we assumed that the diagnosis was RCVS rather than PACNS [11]. Especially, since there was a new ischemic cerebrovascular event in direct temporal association with an up-dosage of prednisolone during the inpatient stay due to severe arthralgias. Prednisolone was slightly reduced by us a few days earlier.

In conclusion, we are the first to present a case showing that glucocorticoids, even at low doses, can lead to RCVS. So far, there are only studies showing that an existing RCVS can be worsened by glucocorticoid administration. Further studies would be interesting to show, whether there is a threshold for the development or worsening of RCVS for glucocorticoids. In our case, prednisolone was necessary due to the relapses in the context of rheumatoid arthritis. Although immunosuppressants and immunomodulators can trigger RCVS, there are no known case reports on TNF-α inhibitors [12]. In contrast, patients taking TNF-α inhibitors for inflammatory bowel disease or rheumatic autoimmune disease frequently developed inflammatory demyelinating (multiple sclerosis, optic neuritis) or non-demyelinating events (meningitis, encephalitis and vasculitis) with mainly leukocytosis and elevated protein levels in CSF as neurological side effects [13, 14]. The use of TNF-α inhibitors may lead to a slight leukopenia in the blood. There have been no reports of such findings in CSF to date [15]. In addition, the patient has been taking the above-mentioned drugs (including TNF-α inhibitor) for many years. There has been an increase in cerebrovascular ischemic events only in the last one and a half years in temporal relation with an increase in prednisolone due to exacerbated arthralgias in the setting of rheumatoid arthritis.

## Data Availability

The data sets used and/or analyzed during the current study are available from the corresponding author on reasonable request.
